# Orthopaedic manifestations and diagnostic clues in children with Guillain–Barré syndrome

**DOI:** 10.1007/s11832-012-0475-2

**Published:** 2013-01-08

**Authors:** Masaki Matsushita, Hiroshi Kitoh, Kazuya Itomi, Takahiko Kitakoji, Koji Iwata, Kenichi Mishima, Naoki Ishiguro, Tadashi Hattori

**Affiliations:** 1 Department of Orthopaedic Surgery, Nagoya University Graduate School of Medicine, 65 Tsurumai, Showa-ku, Nagoya, Aichi 466-8550 Japan; 2 Department of Orthopaedic Surgery, Aichi Children’s Health and Medical Center, Obu, Aichi Japan; 3 Department of Pediatric Neurology, Aichi Children’s Health and Medical Center, Obu, Aichi Japan

**Keywords:** Guillain–Barré syndrome, Children, Diagnostic clue, Limb pain, Gait disturbance, Loss of tendon reflex

## Abstract

**Introduction:**

Guillain–Barré syndrome (GBS) is an acute inflammatory demyelinating polyneuropathy characterized by symmetric limb weakness. Children with GBS sometimes consult the orthopaedists because of limb pain and gait disturbance. The orthopaedists, however, are unfamiliar with GBS, since it has rarely been delineated in detail in the orthopaedic literature. In the present study, we specifically describe orthopaedic manifestations and diagnostic clues in pediatric GBS.

**Methods:**

We reviewed seven patients with pediatric GBS in regard to age, gender, clinical symptoms, department at the first medical consultation, initial diagnosis, physical and laboratory findings, medical interventions, and the latest clinical outcome.

**Results:**

There were five boys and two girls, with a mean age at presentation of 7.2 years. Gait disturbance associated with lower limb pain and weakness was the most frequent early clinical symptom. Among the five patients who presented initially at the orthopaedic department, three were misdiagnosed. Loss of deep tendon reflexes was seen in all patients. Anti-ganglioside antibodies were positive in three and protein levels of cerebrospinal fluid were elevated in five patients. Six patients recovered completely after intravenous immunoglobulin (IVIG) treatment, while one patient who had not undergone IVIG treatment showed minor residual disability.

**Conclusions:**

Acute symmetrical limb pain and gait disturbance associated with loss of tendon reflexes were important clinical manifestations of pediatric GBS. Early diagnosis is essential to prevent delayed recovery, long-term weakness, and permanent functional disabilities.

## Introduction

Guillain–Barré syndrome (GBS) is an acute inflammatory demyelinating polyneuropathy (AIDP) characterized by symmetric limb weakness and loss of tendon reflexes [1]. It is a putative autoimmune disorder presenting commonly after an infectious illness or immunization [2]. Approximately two-thirds of cases are preceded by symptoms of upper respiratory or gastrointestinal tract infection [3]. The most frequently identified infectious agent associated with subsequent development of GBS is *Campylobacter jejuni*, and 30 % of infections were attributed to *C. jejuni* in one meta-analysis, whereas cytomegalovirus has been identified in up to 10 % [4–6]. Also, exposure to influenza via infection or vaccination has been thought to be a common triggering event of GBS [7].

Guillain–Barré syndrome is recognized as a heterogeneous disorder with various clinical manifestations. As well as the classical diagnostic characteristics including ascending weakness and reflex loss, pain and bladder (or bowel) involvement are often features of GBS. Extensive “mimic disorders”, including peripheral neuropathies, disorders of the neuromuscular junction, disorders of muscle, and disorders of the central nervous system, should be excluded in the differential diagnosis of GBS [8–10]. Electrophysiologic examinations play a determinant role in GBS diagnosis, classification of the subtypes, and in establishing prognosis. Recently, different electrodiagnostic criteria have been proposed for AIDP and acute motor axonal neuropathy (AMAN) [11].

Guillain–Barré syndrome is more common in the elderly adult population but rare in children. The incidence of GBS in children less than 17 years old is estimated at 0.8 per 100,000 [12]. In pediatric GBS, not only the rarity of the disease but also limited patient cooperation during neurologic examinations make the diagnosis more difficult, leading to delay in diagnosis or misdiagnosis. Moreover, children with GBS sometimes consult the orthopaedists because of limb pain and gait disturbance [13]. Thus, it is important for orthopaedists to recognize the clinical characteristics and diagnostic clues in pediatric GBS. In the present study, seven children with GBS were retrospectively reviewed, with specific focus on orthopaedic manifestations of the disease.

## Materials and methods

We performed a retrospective study of seven children with the diagnosis of GBS between 2004 and 2009 at the authors’ institution. Medical records and laboratory data were reviewed in regard to age, gender, disease subtype, preceding illness, department at the initial medial consultation, clinical symptoms, physical findings including range of motion (ROM) and manual muscle testing (MMT) of the affected limbs, laboratory findings, initial diagnosis, time to diagnosis from onset or the initial examination, medical interventions, and the latest clinical outcome. On the basis of MMT (grades 0–5) for each muscle, a lower limb strength was calculated as the Medical Research Council (MRC) percentage = (sum of grade scores × 100)/(number of muscles tested × 5) [14]. For the definitive diagnosis, electrophysiologic examinations, including motor conduction studies for the median, ulnar, and tibial nerves and sensory nerve studies for the median and sural nerves, were performed in all patients. Patients were classified as either AIDP or AMAN on the basis of the electrodiagnostic criteria reported by Ho et al. [15]. All patients were followed up for at least 6 months and functional disabilities were graded using the functional grading scale of Hughes et al. [16] at the latest examinations (Table 1).

**Table 1:** Functional grading scale of Hughes et al. [16]

Grade 0	Normal functional state
Grade 1	Able to run with minor signs and symptoms
Grade 2	Able to walk 5 m independently
Grade 3	Able to walk 5 m with assistance
Grade 4	Bed- or chair-bound
Grade 5	Requires assisted ventilation

## Results

Table 2 summarizes the patient data. There were five boys and two girls, with a mean age at presentation of 7.2 years (range 1.5–14.8 years). Two patients had AIDP and five had AMAN. The onset of GBS was preceded by an infectious illness in five patients, including acute gastroenteritis (AGE) in three AMAN and one AIDP patients and upper respiratory infection (URI) in one AIDP patient. These infections occurred between 3 and 10 days before the onset of the disease. There were no prior significant or concomitant diseases in five patients, while one patient had cerebral palsy and the other had autism.

**Table 2:** Characteristics of the patients with Guillain–Barré syndrome (GBS)

Case	1	2	3	4	5	6	7
Age (years)	2.3	1.5	3.5	13.9	14.8	10.3	3.9
Gender	Male	Female	Male	Male	Female	Male	Male
Subtype	AMAN	AIDP	AMAN	AMAN	AMAN	AMAN	AIDP
Preceding illness	None	URI	AGE	AGE	AGE	None	AGE
Distribution of pain	No pain	Bilateral hips	Bilateral legs	Bilateral legs, lower back, neck	Bilateral thighs, back, neck	Bilateral thighs	Neck
MRC percentage of the lower limbs (%)	88	Unevaluable	Unevaluable	68	60	68	84
DTR	Lower limb hyporeflexia	Lower limb hyporeflexia	Lower limb hyporeflexia	Upper and lower limb hyporeflexia	Lower limb hyporeflexia	Lower limb areflexia	Upper and lower limb hyporeflexia
Bladder dysfunction	+	+	−	+	−	−	−
Anti-ganglioside antibody	+	−	+	+	−	−	−
CSF protein (mg/dl)	31.3	181.4	96.4	44.6	23	300	60.4
Initial diagnosis	Irritable hip	GBS	Unknown	Joint pain due to fever	Unknown	Unknown	Meningitis
Duration from onset to diagnosis (days)	10	15	13	7	6	15	4
Department at the first examination	Orthopaedics	Pediatrics	Orthopaedics	Orthopaedics	Orthopaedics	Orthopaedics	Pediatrics
Medical treatment	IVIG	IVIG	None	IVIG	IVIG	IVIG	IVIG
Function at nadir (Hughes et al.’s scale)	3	4	4	3	4	4	4
Final function (Hughes et al.’s scale)	0	0	1	0	0	0	0

*AIDP* acute inflammatory demyelinating polyradiculoneuropathy, *AMAN* acute motor axonal neuropathy, *CSF* cerebrospinal fluid, *URI* upper respiratory infection, *AGE* acute gastroenteritis, *MRC* Medical Research Council, *DTR* deep tendon reflexes, *IVIG* intravenous immunoglobulin

The most frequent early clinical symptom was a gait disturbance, which was associated with lower limb pain or weakness. Pain was evident in six patients during the course of illness. The distribution of pain included lower limb in five, neck in three, and back in two patients. All but two infants for whom discriminative and evaluable MMT had not been measured showed evidence of lower limb weakness, with an MRC percentage of 60–88. Three infants (Cases 2, 3, and 7) refused to stand or walk because of persistent pain of various locations. The other infant (Case 1) without complaint of pain appeared to show significant distress in standing. Three older patients (Cases 4–6), who complained of severe lower limb pain, presented with unsteady crouch gait with their parents’ support. One patient (Case 3) showed 20° of flexion contracture in bilateral knees, while hip ROM was slightly limited (approximately 100° in flexion) in two patients (Cases 2 and 4). Deep tendon reflexes of the lower limbs were partially or completely diminished in all patients. In addition, two patients showed hyporeflexia of the upper extremities. The straight leg raising (SLR) test was positive in one patient (Case 6). Bladder dysfunction such as frequent urination and oliguria was seen in three patients.

Routine blood investigations, including complete blood counts, erythrocyte sedimentation rate, C-reactive protein, and serum creatine kinase, were all within normal values. Magnetic resonance imaging of the brain and total spinal cord demonstrated no evidence of neurological tumors or infection. Serum anti-ganglioside antibodies and cerebrospinal fluid (CSF) were measured in all patients during the treatment period. Anti-ganglioside antibodies that are strongly associated with the AMAN form were positive in three of the five AMAN patients. CSF examination revealed elevated levels of protein in five patients and normal levels in two. Electrophysiologic examinations showed various types of abnormalities in the peripheral nerve conduction, including prolonged distal motor latency, reduced motor nerve conduction velocity, reduced compound muscle action potentials (CMAP), and reduced sensory nerve action potentials (SNAP). AIDP patients showed longer distal motor latencies and slower nerve conduction velocities than AMAN patients. Reduced CMAP amplitudes were observed in both subtypes, whereas SNAP amplitudes were almost normal in AMAN and decreased in AIDP patients.

**Fig. 1:**
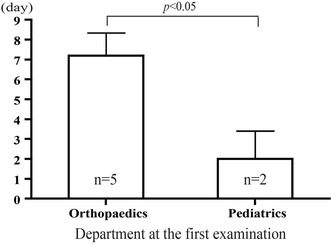
The average time to diagnosis from initial medical consultation was 7.2 days in patients who presented to the orthopaedists and 2.0 days in those who presented to the pediatricians. There was a significant association between the diagnostic delay and the department at the initial consultation (*P* < 0.05). The results are expressed as mean + standard deviation (SD). Statistical analysis was carried out using Student’s *t*-test, with significance set at *P* < 0.05

All patients were finally diagnosed as GBS by the pediatric neurologists based on the current diagnostic criteria of GBS [1]. (1) Features required for diagnosis: progressive motor weakness of more than one limb and areflexia (loss of tendon jerks). (2) Features strongly supportive of the diagnosis: progressive motor weakness, relative symmetry, mild sensory symptoms, cranial nerve involvement, recovery of the symptoms, autonomic dysfunction, absence of fever at the onset, elevated CSF protein and cells, and electrodiagnostic abnormalities. (3) Features casting doubt on the diagnosis: persistent asymmetry of weakness, bladder or bowel dysfunction, elevated mononuclear leukocytes in CSF, presence of polymorphonuclear leukocytes in CSF, and sharp sensory level. Three patients were misdiagnosed as irritable hip, meningitis, and joint pain due to fever, respectively. Initial diagnoses had not been done in three patients. Only one patient was diagnosed as GBS at the first examination by the pediatric neurologist. The average times to correct diagnosis from onset and from initial medical consultation were 10.0 days (range 4–15 days) and 5.7 days (range 0–11 days), respectively. Five patients initially consulted the orthopaedists, while the other two patients consulted the pediatricians. The diagnostic delay was statistically significant in patients who were examined first at the orthopaedic department (Fig. 1).

Six patients who received intravenous immunoglobulin (IVIG) treatment immediately after the diagnosis of GBS demonstrated complete recovery of neurologic functions (Hughes et al.’s scale: 0) after treatment. Glucocorticoid treatment was done in one patient before IVIG therapy. One patient (Case 3) who was already in the recovery process when definitive diagnosis was made, did not receive IVIG therapy because the pediatric neurologists concluded the therapy to be less effective. He showed limited range of bilateral ankle dorsiflexion of 5° at the latest examination (Hughes et al.’s scale: 1).

## Discussion

Guillain–Barré syndrome is a recognizable entity for the commonest cause of acute childhood flaccid paralysis in the field of pediatric neurology [9]. Several reports in larger numbers of GBS patients have been delineated from the neurologists’ point of view, but there are few reports which specifically describe orthopaedic aspects [17, 18]. Some of the pediatric GBS patients consult the orthopaedists first because limb pain and gait disturbance are frequent primary complaints. Indeed, five of the seven patients initially consulted the orthopaedic department in the present study. The orthopaedists, however, are unfamiliar with the disease, since it has rarely been reported in detail in the orthopaedic literature. To prevent diagnostic delay, orthopaedists should be aware of the early symptoms and clinical manifestations of pediatric GBS.

Nguyen et al. [9], reviewing a series of GBS patients under 6 years old, showed that pain was a primary complaint and, sometimes, preceding the onset of paralysis. Various types of pain including deep aching pain, throbbing pain, and radiation pain have been described. Moulin et al. [19] stated that acute back and leg pain commonly exacerbated by SLR provide indirect evidence that traction on inflamed nerve roots could be responsible for some of the pain. In the present study, all but one patient complained of pain during the course of illness, and symmetrical lower limb pain was present more specifically in five patients. Limb weakness, which is caused by conduction block in motor nerves and leads to a gait disturbance, is another characteristic finding in GBS. Due to limb weakness, three patients over 10 years of age could not walk independently and four infants under 4 years of age refused to walk or stand in our study. In addition, loss of tendon reflexes, which was confirmed in all patients in the present study, seemed to be a universal neurological finding in GBS. Therefore, further laboratory examinations of GBS should be taken into consideration for the patients presenting with acute limb pain and gait disturbance associated with hyporeflexia or areflexia.

Electrophysiologic findings are the most sensitive and specific in GBS [20]. We finally confirmed various abnormalities of peripheral nerve conductions in all patients. Similar to the report of Nagasawa et al. [21], the majority of our patients showed AMAN form and three of them showed positive anti-ganglioside antibodies. In addition, most of our patients demonstrated elevated levels of CSF protein that is commonly seen in pediatric GBS. Ropper [10] noted that a protein concentration above 0.55 mg/dl was a confirmatory finding in fully developed GBS. The precise diagnosis of GBS can be made based on these laboratory examinations, as well as characteristic clinical manifestations.

Patients not receiving appropriate treatments sometimes provide unsatisfactory outcome, such as delayed recovery and long-term weakness. IVIG and plasmapheresis halt the progression of GBS and shorten recovery time. IVIG treatment, which has anti-inflammatory properties at the nerve root level, is preferred in childhood because of less invasive and rarer complications than plasmapheresis. Hughes et al. [22] described that IVIG treatment significantly shortened the duration of symptoms and reduced disease severity when started early in the course of the disease. Indeed, six of our patients recovered completely within 6 months after IVIG treatment. Nagasawa et al. [21], on the other hand, reported that appropriate treatment was done in only 61 % of children aged 3 years or younger, probably due to difficulties in the early diagnosis of pediatric GBS. One patient who had not undergone IVIG treatment remained having minor residual disability in the ankle ROM in this series. Early diagnosis, therefore, is essential to prevent delayed recovery, long-term weakness, and permanent functional disabilities.

**Fig. 2:**
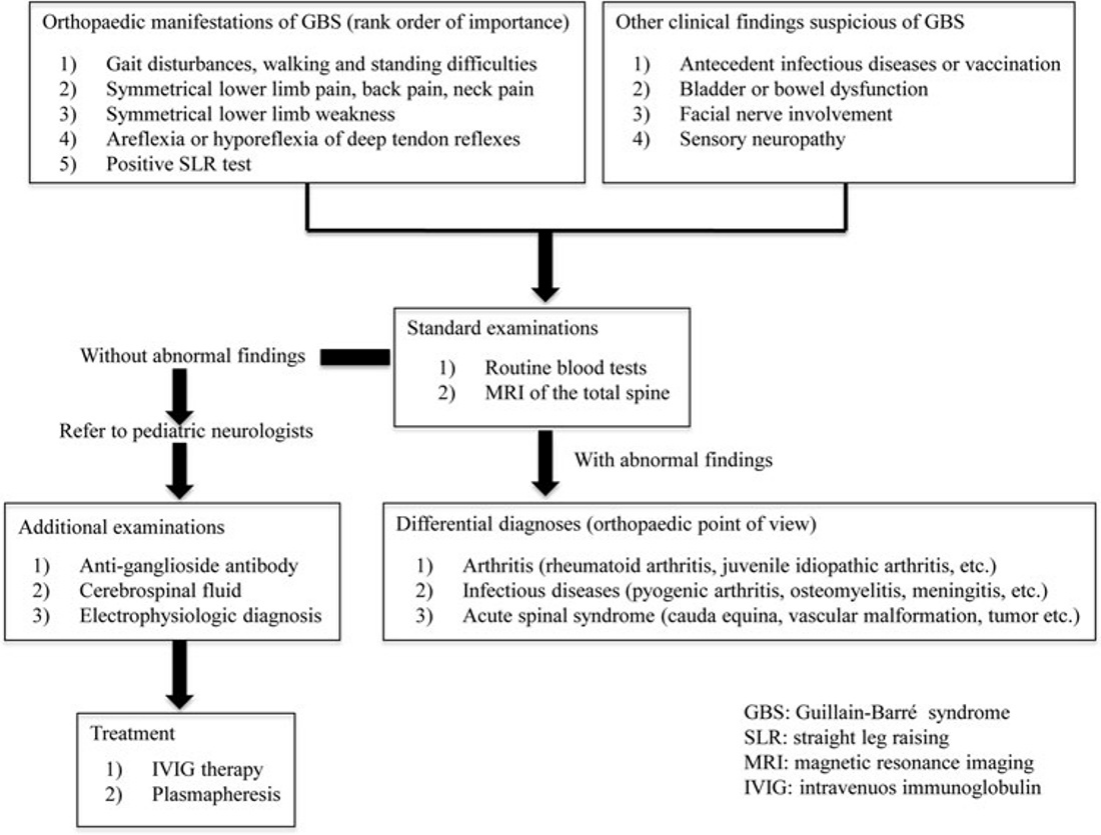
Orthopaedic manifestations and diagnostic clues in pediatric patients with Guillain–Barré syndrome

In conclusion, orthopaedic manifestations of pediatric GBS include: (1) gait disturbances, walking and standing difficulties; (2) symmetrical lower limb pain, back pain, and neck pain; (3) symmetrical lower limb weakness; (4) areflexia or hyporeflexia of deep tendon reflexes; and (5) less frequently positive SLR test (Fig. 2). Thus, GBS should be considered in the differential diagnosis of any child presenting with acute limb pain and weakness associated with loss of deep tendon reflexes. Orthopaedists should refer to the pediatric neurologists for definitive diagnosis and prompt treatment when other musculoskeletal diseases analogous to GBS are excluded.
